# Coping and metabolic syndrome indicators in urban black South African men: the SA BPA study

**DOI:** 10.5830/CVJA-2010-024

**Published:** 2010

**Authors:** A Du Plessis, L Malan, NT Malan

**Affiliations:** School for Physiology, Nutrition and Consumer Sciences, North-West University, Potchefstroom, South Africa; School for Physiology, Nutrition and Consumer Sciences, North-West University, Potchefstroom, South Africa; School for Physiology, Nutrition and Consumer Sciences, North-West University, Potchefstroom, South Africa

**Keywords:** Africans, urban, coping, metabolic syndrome, hypertension, obesity

## Abstract

Urbanisation is associated with obesity, hypertension and development of the metabolic syndrome (MS). We aimed to assess the use of different coping styles and their influence on increases in MS indicators and target end-organ damage (TOD) in urban black African men. A sample of 53 men was classified as clear high active (AC, *n* = 30) or passive coping (PC, *n* = 23) responders, using the Amirkhan African validated coping style indicator. Blood pressure (BP) was recorded with an aneroid sphygmomanometer and waist circumference (WC) was determined. Carotid intima–media thickness (CIMT) and microalbuminuria were analysed to determine TOD.

Fasting serum and eight-hour urine samples revealed elevated MS indicators in AC men. Strong associations existed between MS indicators and TOD in AC but not PC men. To conclude, only BP and seeking social support were positively associated with TOD in urban PC African men, while in urban AC African men, most MS indicators were positively associated with TOD, i.e. sub-clinical atherosclerosis and renal impairment.

## Summary

The World Heart Federation stated in 2006 that obesity is associated with type 2 diabetes, hypertension and the metabolic syndrome (MS), all of which cause cardiovascular disease (CVD).^1^ As obesity is on the increase in South Africa, especially in urban areas, with up to 45% of the population already overweight, the prevalence of the MS is increasing.^2^ The THUSA study (Transition and Health during Urbanisation in South Africa) revealed a greater prevalence of CVD and MS indicators in urban black South Africans (hereafter referred to as Africans).^3^ Furthermore, Malan *et al*.^3^-^5^ revealed that urban African men utilising an active coping (AC) style were at an increased risk for hypertension, the MS and increased blood glucose values.

Van Rooyen *et al*. stated that the BP of Africans changes proportional to their age, level of urbanisation, and waist-to-hip ratio.^6^ There is a high prevalence of hypertension in African-Americans.^7^ This is mostly due to increased sodium retention, dietary deficiencies and augmented socio-economic stress associated with westernisation.^7^ Analogous with the aforementioned, black adults in most industrialised societies have extraordinarily high hypertension rates, which are probably due to increased salt sensitivity in this population group.^8^

Contrasting literature also exists relating to specific coping styles and their influence on cardiovascular status. Malan *et al*. indicated in 2008 that urban Africans utilising an active coping style were at an increased risk for hypertension and the MS.3-5 This was opposed to van Rhenen *et al.* who stated that utilising an AC style promotes health.^9^

In accord with van Rhenen, Obrist stated that an AC style is associated with problem solving, perception of control and central cardiac β-adrenergic response patterns [increases in systolic (SBP) and diastolic blood pressure (DBP), as well as cardiac contractility and output].^10^ A PC style, on the other hand, is rather associated with hopelessness and depression, indicating loss of control and increases in vascular α-adrenergic response patterns [e.g. DBP, peripheral resistance (TPR), with subsequent decreases in arterial compliance].^10^ In 2004, it was rather a PC style, an avoidant strategy, which correlated positively with blood pressure levels, which in turn was correlated with pathology 11,12

According to the International Diabetes Federation (2006), there are five cardiovascular risk factors underlying the development of the metabolic syndrome. These are abdominal obesity, diabetes, high blood pressure, and high levels of blood glucose and high-density lipoprotein (HDL).^13^ Nevertheless, sedentary lifestyle, higher dietary fat intake and psychosocial stress are also major contributing factors in the development of the MS.^14^

Atherogenic dyslipidaemia is generally observed in MS patients.^13^-^17^ A combination of elevated serum triglycerides and apolipoprotein B, together with reduced HDL levels, and increased VLDL (very low-density lipoprotein) values is typically reported.^12^ Dyslipidaemia is associated with coronary heart disease and progression of target end-organ damage, and its levels increase with lower socio-economic status in developing countries.^18^

In the present study, the main aim was to assess whether a relationship could be observed between individual risk factors that may constitute the MS in urban African men, and the utilisation of specific coping styles. The hypotheses are then, firstly, that utilising an AC style will be associated with negative changes in individual indicators of the MS in urban African men, and secondly, that the changes in MS indicators will be associated with target end-organ damage, depicted by carotid intima–media thickness and microalbuminuria.

## Methods

The SABPA study (Sympathetic Activity and Ambulatory Blood Pressure in Africans), a target population study on black South African teachers, was conducted from February to May 2008. It included 101 recruited urban black African males aged between 25 and 60 years, with the same socio-economic status, and complying with the inclusion criteria. They were selected from one of the four Dr Kenneth Kaunda education districts of the North West province, South Africa. To assimilate the sample, the following exclusion criteria were proposed: users of α- and β-blockers, participants with body temperatures higher than 37.5°C, and those who had been vaccinated or had donated blood three months prior to participation.

For the purpose of our sub-study, we included only 53 men of the 101 SABPA black males after they had been classified as clear high active (AC, *n* = 30) or passive coping (PC, *n* = 23) responders, using the Amirkhan African validated coping style indicator.^19^

The North West Education Department supported by the South African Democratic Teachers Union granted permission for participation in the study. The Ethics Committee of the North-West University approved the study and all the participants completed informed consent forms prior to cooperation. We abided by the institutional guidelines and terms of the Declaration of Helsinki when taking measurements and conducting procedures.^20^

## Experimental procedure

At 07:00 on four working days of the week, application of the Cardiotens® for 24-hour blood pressure measurements and the Actical® apparatuses commenced at their respective institutions. Teachers thereafter resumed their normal daily activities and at the end of the day, were transported to the North West University’s research facility to overnight. They were each welcomed, received their own rooms and were pre-counselled regarding AIDS. Afterwards they were exposed to the experimental set up to lessen anticipatory stress.^10^

Completion of the psychosocial questionnaires followed, supervised by registered clinical psychologists, with a dinner break in between, and participants went to bed at 22:00, fasting overnight. At 06:00 the following morning, disconnection of the Cardiotens® occurred and an eight-hour collected fasting urine sample was obtained from each volunteer. Anthropometric measurements began, followed by a resting period of five to 10 minutes, and subsequently, blood sampling commenced.

On completion of the protocol, the participants were thanked for their cooperation and each received feedback and post-counselling for HIV (if tested positive) in the privacy of their rooms. Transportation back to school followed breakfast and referral to a physician, where applicable.

## Questionnaires

The coping strategy indicator (CSI), developed by Amirkhan (1990), was essential in predicting each participant’s predominant or habitual coping style.^21^ The CSI is a self-report measure of coping strategies, encompassing problem and avoidance strategies, and seeking social support. This 33-item questionnaire, formulated through a combination of deductive and inductive methodologies, is widely applicable and has been validated for Africans.^21^

Participants rated the 33 items of the questionnaire on a threepoint Likert scale: a lot (3), a little (2), or not at all (1), with a recent stressful event in mind. The higher scores were indicative of preference for a specific coping style, with the cut-off points for high use pertaining to: problem solving or AC (31), avoidance or PC (23), and seeking social support (28).^21^ Clear high responders were scrutinised,^21^ and participants utilising both AC and PC styles (*n* = 48) were excluded. Unfortunately this could not be prevented and was a limitation of the sub-study.

The global physical activity questionnaire (GPAQ) as predictor of the subjects’ physical activity was completed and assessed.^22^ Participants also completed the general socio-demographic and health questionnaire regarding information such as medical history, alcohol and smoking habits, caffeine intake, and sociodemographic details.

## Anthropometrics

Registered level II biokinetisists took measurements of each participant in triplicate to ensure accuracy. Measurement of waist circumference (WC) was perpendicular to the long axis of the trunk, at the midpoint between the lower costal border and iliac crest. The cut-off points for WC determining obesity were values of ≥ 94 cm (male) and ≥ 80 cm (female).^13^ The BMI was calculated in kg/m^2^ from the height and weight.

Actical® accelerometers (Mini Mitter Co, Bend, Oregon, USA) on the waist measured physical activity (PAI) in kilocalories per 24 hours, and participants were classified as high activity (PAI-3) according to their active energy expenditure. PAI-3 was equivalent to strenuous activity for three days a week (achieving a minimum of 1 500 MET-minutes), or any activity accruing at least 3 000 MET-minutes for seven days a week.^22^

## Blood pressure

The Cardiotens® (Meditech, Budapest, Hungary) apparatus obtained 24-hour blood pressure measurements. This programmed apparatus measures ambulatory blood pressure oscillometrically at intervals of 30 minutes during the day and 60 minutes at night. A suitable obese or non-obese cuff of the Cardiotens® apparatus was fastened to each subject’s non-dominant arm.

After overnight sleep and anthropometrical measurements, two mercury sphygmomanometer blood pressure readings using Korotkoff IV or V for diastolic BP followed while the partici- pants rested for five minutes in the semi-fowler position, with a three- to five-minute rest in between measurements. Participants were defined as hypertensive with a 24-hour blood pressure of > 125–130/> 80 mm Hg, according to the ESH guidelines (2007).^23^

## Carotid intima–media thickness (CIMT)

A high-resolution ultrasound scan with CIMT images from at least two optimal angles of the left and right common carotid artery, carotid bulb and internal carotid arterial (ICA) segments were obtained using a Sonosite Micromaxx ultrasound system (SonoSite Inc, Bothell, WA, USA) and 6–13 MHz linear array transducer using the Rudy Meijer protocol. The digitised images were imported into the AMS automated software for analysis of CIMT. A maximal 10-mm segment with good image quality was chosen for analysis. The program automatically identifies the borders of the CIMT of the near and far wall.

## Biochemical analysis

Fasting resting serum and sodium fluoride (glucose) blood samples were obtained by a registered nurse from the brachial vein branches of the participant’s dominant arm, with a winged infusion set. Blood samples were centrifuged at 3 700 revolutions per minute for 10 minutes, separated and frozen at –80°C until analysis.

An overnight (eight-hour) collected fasting urine sample was used for the assessment of microalbuminuria to indicate target end-organ damage (TOD). Measurement of albumin excretion followed by means of immune precipitation, enhanced by polyethylene glycol at 450 nm (reference range of creatinin:albumin ratio = 0–2.9 mg/µmol^17^). The sequential multiple analyser computer (Konelab™, Vantaa, Finland) calculated fasting glucose, triglycerides, cholesterol and HDL levels.

HIV/AIDS screening was done with antibody tests, namely the First Response® kit (RPM Plus, Colonia, New Jersey, USA) and the Pareekshak test (Bhat Biotech, India) to determine the participants’ status.

## Statistical analysis

Data were analysed using the Statistica 8.0 software (Statsoft Inc, Tulsa, USA). Normal distributions of the variables determined with the Kolmogorov-Smirnov test revealed symmetrical data, but microalbuminuria data were not evenly distributed and therefore logarithmic transformation was performed. *T*-tests followed to determine significant differences in age, BMI, alcohol consumption, smoking and PAI between coping style groups. Chi-square tests (χ^2^) determined the prevalence of each factor. Analysis of covariance (ANCOVA), adjusting for alcohol consumption as co-factor, was used to compare MS data between AC and PC African men.

According to the International Diabetes Federation (2006), central obesity should be present together with two or more of the following factors, in order to diagnose the MS: increased triglyceride levels (> 1.7 mmol/l); reduced HDL cholesterol (< 1.03 mmol/l in men and < 1.29 mmol/l in women); raised fasting plasma glucose concentrations (> 5.6 mmol/l); and hypertension (> 130/> 80 mmHg).^13^

Partial correlations (adjusted for alcohol) were performed between MS indicators and markers of target end-organ damage. Multiple regression analyses using MS indicators as independent variables were performed to explain microalbuminuria and carotid intima–media thickness of the far wall (CIMT_f_). For CIMT_f_, additional adjustments for BP and lipids were done. Significant values were noted as *p* ≤ 0.05, *r* ≥ 0.35, and adjusted *r*^2^ ≥ 0.25.

All statistical analyses, i.e. analysis of covariance and partial correlations were repeated after exclusion of participants treated with hypertensive, diabetic and statin medications. No significant differences prevailed and therefore discussion on these results is not warranted.

## Results

In the SABPA study, 53 urban black African men were included and stratified into AC and PC groups. Table 1 compares the characteristics of the African men and MS indicators of the urban AC and PC men. Only PC men showed higher alcohol consumption and cholesterol values.

**Table 1.** Descriptive Statistics And Ancovas Compared AC And PC Characteristics Including Ms And Target End-Organ Damage Indicators, Independent Of Alcohol Consumption (Mean ± 95% Ci)^19^

	*Urbanised men (n = 101)*		
	*Active coping (n = 30)*	*Passive coping (n = 23)*	p*-value*
Age (years)	41.77 (34.59, 48.94)	41.52 (34.22, 48.82)	0.90
Body mass index (kg/m^2^)	28.08 (22.06, 34.11)	27.08 (22.97, 31.19)	0.50
Alcohol, *n* (%)*	**7 (23.33)^a^**	**12 (52.17)^a^**	**0.03**
Smoking, *n* (%)*	8 (26.67)	8 (34.78)	0.52
High physical activity, *n* (%)*	3 (10.00)	1 (4.35)	0.56
AIDS, *n* (%)*	5 (16.67)	2 (8.70)	0.40
Anti-hypertensive drugs, *n* (%)*	**4 (13.33)^b^**	**0 (0.00)^b^**	**0.07**
Anti-diabetic drugs, *n* (%)*	1 (3.33)	0 (0.00)	0.38
Social support, *n* (%)*	14 (46.67)	8 (34.78)	0.38
Cholesterol (mmol/l)^§^	**4.36 (3.96, 4.76)^c^**	**5.09 (4.63, 5.55)^c^**	**0.02**
Systolic BP (mmHg)^§^	138.33 (131.72, 144.94)	133.48 (125.88, 141.08)	0.35
Diastolic BP (mmHg)^§^	88.68 (84.57, 92.79)	84.20 (79.48, 88.92)	0.17
MS indicators			
Hypertension, *n* (%)*	23 (76.67)	15 (65.22)	0.36
Waist circumference (cm)^§^	93.65 (88.56, 98.74)	92.68 (86.83, 98.53)	0.81
Gucose (mmol/l)^§^	6.09 (5.33, 6.85)	5.40 (4.53, 6.27)	0.25
Triglycerides (mmol/l)^§^	1.60 (1.14, 2.05)	1.51 (0.99, 2.04)	0.81
HDL (mmol/l)^§^	1.04 (0.91, 1.17)	1.07 (0.92, 1.22)	0.74
Target end-organ damage			
Microalbuminuria (µg/l/min)^§^	***1.04 (0.92, 1.16)^d^***	***0.87 (0.73, 1.01)^d^***	***0.08***
CIMT_f_ (mm)^§^	0.68 (0.62, 0.74)	0.71 (0.64, 0.78)	0.49

^§^ ANCOVA: analysis of covariance; MS: metabolic syndrome; AC: active coping; PC: passive coping; ± 95% CI: 95% confidence intervals; n: number of participants; independent *t*-test values; *Chi-squares; HDL: high-density lipoproteins; CIMT_f_: carotid intima–media thickness far wall. Values in bold with identical alphabetical superscripts differ significantly, *p* ≤ 0.05, and in bold italics are borderline significant.

**Fig. 1.**
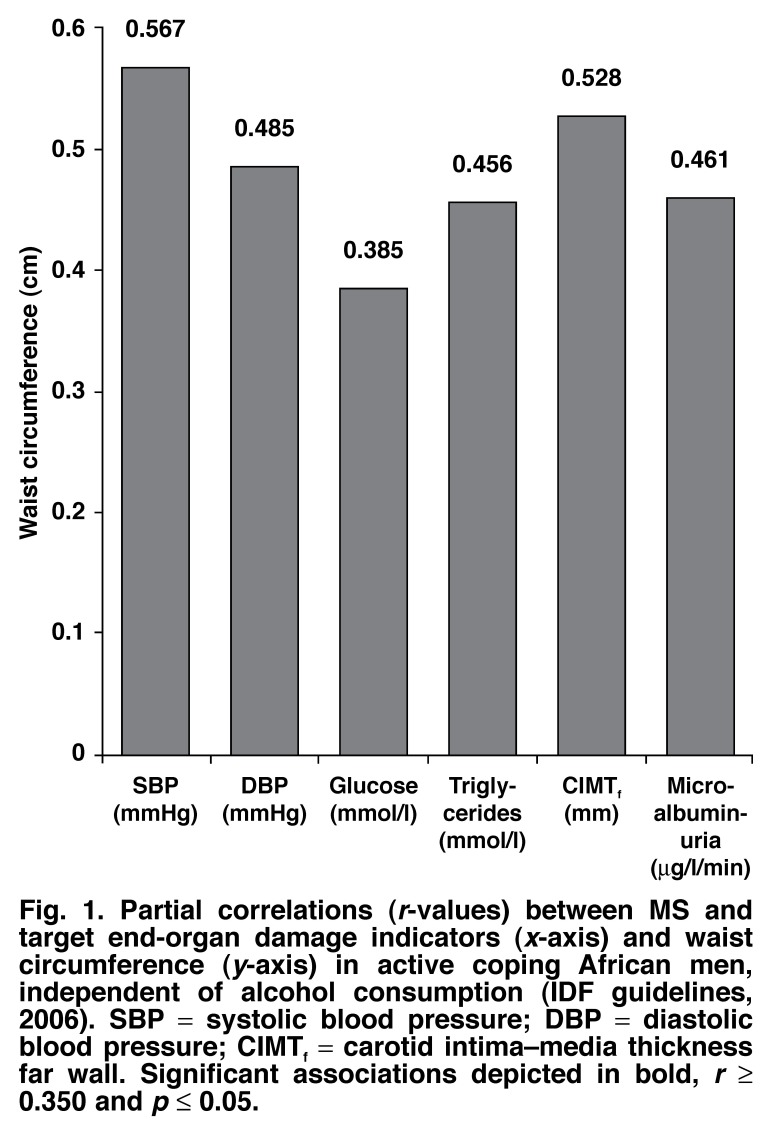
Partial correlations (*r*-values) between MS and target end-organ damage indicators (*x*-axis) and waist circumference (*y*-axis) in active coping African men, independent of alcohol consumption (IDF guidelines, 2006). SBP = systolic blood pressure; DBP = diastolic blood pressure; CIMT_f_ = carotid intima–media thickness far wall. Significant associations depicted in bold, *r* ≥ 0.350 and *p* ≤ 0.05.

**Fig. 2.**
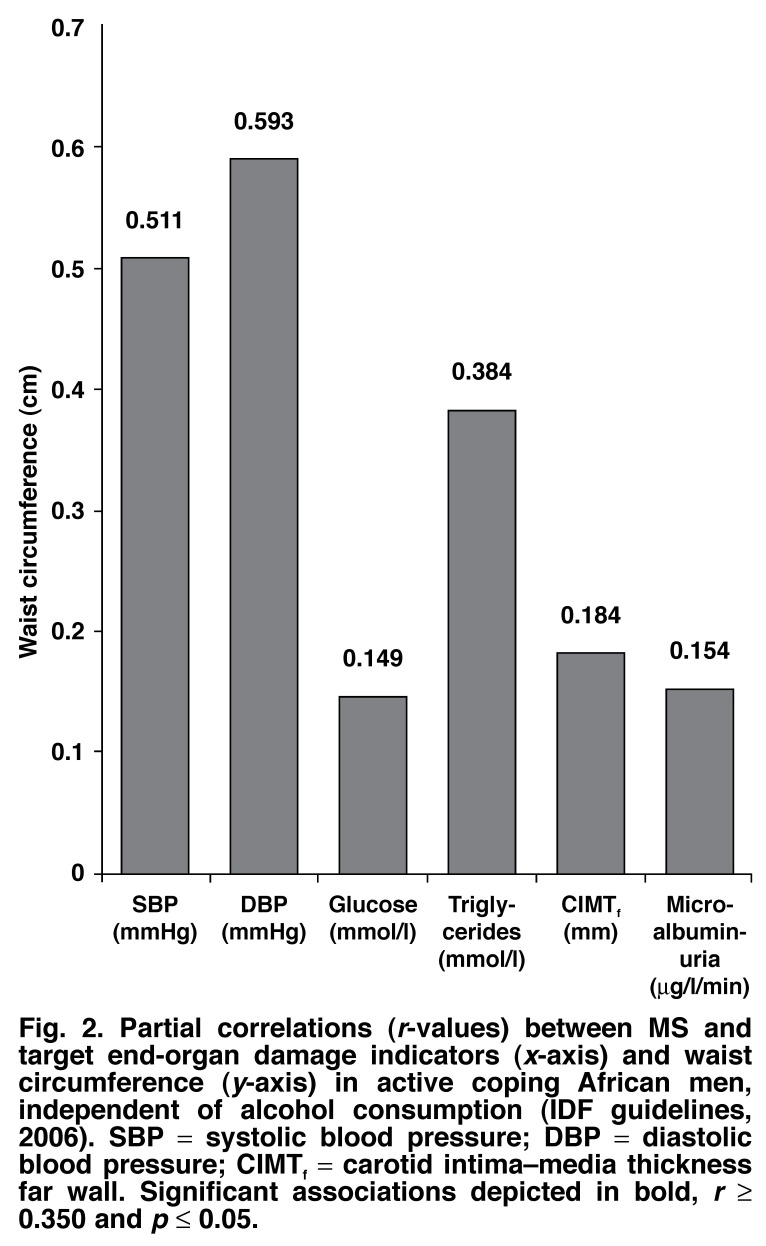
Partial correlations (*r*-values) between MS and target end-organ damage indicators (*x*-axis) and waist circumference (*y*-axis) in active coping African men, independent of alcohol consumption (IDF guidelines, 2006). SBP = systolic blood pressure; DBP = diastolic blood pressure; CIMT_f_ = carotid intima–media thickness far wall. Significant associations depicted in bold, *r* ≥ 0.350 and *p* ≤ 0.05.

In Fig. 1, in AC men, waist circumference, as an essential prerequisite of the MS (IDF, 2006), correlated positively (*p* ≤ 0.05) with SBP (*r* = 0.57), DBP (*r* = 0.49), glucose (*r* = 0.39) and triglyceride levels (*r* = 0.46). Additionally, highly significance correlations (*p* ≤ 0.05) were observed in AC men between WC and TOD, respective correlations for microalbuminuria (*r* = 0.46) and CIMT_f_ (*r* = 0.53). However, in PC men (Fig. 2), correlations existed between only WC and blood pressure.

In Table 2, in AC men, the HDL levels correlated significantly (*p* ≤ 0.05) with SBP (*r* = –0.45), DBP (*r* = –0.40) and CIMT_f_ (*r* = –0.49). Strong correlations (*p* ≤ 0.05) also existed between microalbuminuria and glucose (*r* = 0.50) as well as triglyceride levels (*r* = 0.56). Another significant (*p* ≤ 0.05) correlation existed between glucose levels and CIMT_f_ (*r* = 0.63). In Table 3, in PC men, HDL and triglyceride levels correlated significantly (*r* = –0.53; *p* ≤ 0.05). Correlations also existed between microalbuminuria and SBP as well as DBP, with significant *p*-values (*p* ≤ 0.05).

**Table 2.** Partial Correlations Of MS And Target End-Organ Damage Indicators In Urban Active Coping Black Men, Independent Of Alcohol Consumption^19^

	*Active coping (n = 30)*									
	*SBP (mmHg)*		*DBP (mmHg)*		*Glucose (mmol/l)*		*TG (mmol/l)*		*CIMT_f_ (mm)*	
	r	p	r	p	r	p	r	p	r	p
Glucose (mmol/l)	***0.324***	***0.086***	0.273	0.152	1.000	–	***0.301***	***0.070***	**0.633**	**0.000**
TG (mmol/l)	0.215	0.201	0.121	0.533	0.243	0.203	1.000	–	0.215	0.262
HDL (mmol/l)	**–0.455**	**0.013**	**–0.402**	**0.031**	–0.263	0.169	–0.262	0.171	**–0.488**	**0.007**
WC (cm)*	–	–	–	–		–	–	–	–	–
Microalbuminuria (µg/l/min)	0.065	0.736	–0.022	0.911	**0.499**	**0.006**	**0.564**	**0.001**	***0.348***	***0.065***

MS: metabolic syndrome; *n*, number of subjects; SBP: systolic blood pressure; DBP: diastolic blood pressure; TG: triglycerides; HDL: highdensity lipoproteins; CIMT_f_: carotid intima–media thickness far wall. All values in bold with *r* ≥ 0.350 and *p* ≤ 0.05 differ significantly, and values in bold italics are borderline significant. *WC: waist circumference data presented in Fig. 1.

**Table 3.** Partial Correlations Of MS And Target End-Organ Damage Indicators In Urban Passive Coping Black Men, Independent Of Alcohol Consumption^19^

	*Passive coping (n = 23)*									
	*SBP (mmHg)*		*DBP (mmHg)*		*Glucose (mmol/l)*		*TG (mmol/l)*		*CIMT_f_ (mm)*	
	r	p	r	p	r	p	r	p	r	p
Glucose (mmol/l)	0.097	0.676	0.207	0.368	1.000	–	0.188	0.414	–0.044	0.850
TG (mmol/l)	0.083	0.720	0.279	0.221	0.188	0.414	1.000	–	–0.259	0.257
HDL (mmol/l)	–0.103	0.658	–0.096	0.679	–0.164	0.478	**–0.525**	**0.014**	0.124	0.591
WC (cm)*	–	–	–	–	–	–	–	–	–	–
Microalbuminuria (µg/l/min)	**0.773**	**0.000**	**0.710**	**0.000**	0.137	0.554	–0.007	0.977	0.207	0.368

MS: metabolic syndrome; *n*, number of subjects; SBP: systolic blood pressure; DBP: diastolic blood pressure; TG: triglycerides; HDL: highdensity lipoproteins; CIMT_f_: carotid intima–media thickness far wall. All values in bold with *r* ≥ 0.350 and *p* ≤ 0.05 differ significantly, and values in bold italics are borderline significant. *WC: waist circumference data presented in Fig. 2.

In Table 4, multiple regression analyses revealed that an AC style in urban African men was associated with most MS indicators, as well as TOD, depicted by CIMT_f_ and microalbuminuria. Furthermore, in PC men, significant associations existed between social support (*r* = 0.531) and augmented CIMT_f_ values, whereas SBP (*r* = 0.596) and lower WC (*r* = –0.408) were associated with microalbuminuria.

**Table 4.** Multiple Forward Stepwise Regression Analyses Indicating Independent Associations Between Measures Of MS And Target End-Organ Damage Indicators

	*Urban AC African men (n = 30)*		*Urban PC African men (n = 23)*	
	*CIMT_f_ (mm)*	*Microalb (µg/l/min)*	*CIMT_f_ (mm)*	*Microalb (µg/l/min)*
Adjusted r^2^	0.547	0.606	0.588	0.665
Independent variables	β (SE)	β (SE)	β (SE)	β (SE)
Age (years)	–	–	**0.627 (0.15)^§^**	–
Body mass index (kg/m^2^)	–	–	–	–
Alcohol (%)	–	–	0.513 (0.15)*	–
Smoking (%)	–	–	–	–
PAI-3 (kcal/24 h)	–	0.404 (0.13)*	–	–
Social support	–	–	0.531 (0.14)*	–
Cholesterol (mmol/l)	0.407 (0.17)**	–	–	–
SBP (mmHg)	–	–	–	0.596 (0.26)**
DBP (mmHg)	–	–0.360 (0.14)**	–	0.442 (0.28)
Waist circumference (cm)	–	0.789 (0.22)^§^	–	–0.408 (0.16)*
Glucose (mmol/l)	**0.482 (0.14)^§^**	0.354 (0.13)*	–0.282 (0.16)	–
Triglycerides (mmol/l)	–0.236 (0.16)	0.378 (0.13)*	–	–
HDL (mmol/l)	**–0.554 (0.16)^§^**	***0.373 (0.18)***	–	–

MS: metabolic syndrome indicators; AC: active coping; *n*: number of participants; CIMT_f_: carotid intima–media thickness far wall; Microalb: microalbuminuria log transformed; β: beta coefficient, SE: standard error; PAI-3: high physical activity; SBP: systolic blood pressure; DBP: diastolic blood pressure; HDL: high-density lipoproteins. Values in bold and with the same superscripts differ significantly: ^§^*p* ≤ 0.004; *p ≤ 0.01; **p ≤ 0.05, and values in bold italics are borderline significant.

## Discussion

The main purpose of this sub-study was to determine whether different coping styles were associated with MS indicators in urban African men, as sedentary lifestyles, dietary intake and psychosocial stress are on the increase.^14^ Higher levels of MS indicators existed in AC participants in relation to their PC counterparts, according to the IDF guidelines (2006).^13^ These findings corroborate the conclusion of the THUSA study, in which urban AC men were at high risk for development of the MS.^3^

In the abovementioned study, the adoption of an AC style was stated to be a cardiovascular risk factor and this was confirmed in the SABPA study.^3^ Our results revealed a higher prevalence rate of hypertension of 76% in AC men, compared to 65% in PC men.^23^ Opie (2004) stated that utilising a PC style was associated with higher prevalence of hypertension as well as strong positive associations with renal impairment, which is contradictory to our findings.^11^ The results of the PC men however, ultimately did not show associations with MS indicators, only with BP. Nonetheless, utilising an AC strategy correlated positively with SBP, DBP and TPR, together with MS indicators.^3^,^10^

According to the IDF criteria,^13^ the AC group showed higher MS indicators, including increased fasting glucose concentrations of 6.09 mmol/l (AC) and 5.40 mmol/l (PC), respectively. Interestingly, in the AC group, 60% of the men had increased fasting plasma glucose concentrations exceeding the cut-off points, according to the IDF guidelines (≥ 5.6 mmol/l).^13^ Furthermore, 61.5% of the AC men had a WC indicative of abdominal obesity, and this created great concern because of its mentioned pathological effects in the development of the MS and risk of cardiovascular disease.^13^,^24^

Abdominal obesity is the essential prerequisite for diagnosis of the MS, but no group had values significantly higher than 94 cm.^13^ Waist circumference was used to determine abdominal obesity in this study, as BMI analysis is not indicative of heart disease, whereas WC has a positive predictive value for identifying insulin resistance.^25^,^26^ WC was strongly associated with increased SBP, DBP, triglyceride and fasting glucose levels in the AC men, showing a trend towards development of the MS and cardiovascular risk. Conversely, no significant indications existed in the PC men.

The proposed mechanism for established MS could be that visceral adiposity is associated with impaired glucose tolerance and atherogenic dyslipidaemia, which is a combination of increased triglyceride and reduced HDL levels as well as LDL particles.^13^,^15^ As intra-abdominal fat is highly lipolytic and increases fatty acid transport to the liver, a decrease in insulin clearance is inevitable, causing hyperinsulinaemia.^17^ Furthermore, both visceral obesity and hyperinsulinaemia are associated with increased sympathetic activity, favouring re-absorption of Na^+^.^17^ Increased circulating Na+ causes vasoconstriction, and hypertension follows.^15^,^17^

Dually noted, Africans are usually salt sensitive and reveal sympathetic over-activity, and this could probably be the link between the aforementioned mechanism and augmented prevalence of hypertension in Africans.^11^,^27^ As the AC men were not centrally obese, the latter explanation of inherent enhanced sympathetic activity^11^ and subsequent increased MS indicators could rather contribute to the strong associations found between dyslipidaemia, impaired fasting glucose and sub-clinical atherosclerosis, as well as renal impairment.

The HDL findings in AC men are of major significance, as HDL levels correlated negatively with blood pressure and sub-clinical atherosclerosis, which is indicative of atherogenic dyslipidaemia, found in both type 2 diabetes and the MS.^13^ Ultimately, dyslipidaemia could result in coronary heart disease, supporting the fact that the MS and its underlying factors are the most important risk factors for myocardial infarction.^13^,^18^ The lower cholesterol values in AC men compared to PC men were positively associated with CIMTf, which is difficult to explain, as this clearly contradicts literature regarding the role of cholesterol and development of sub-clinical atherosclerosis^11^,^13^,^16^-^18^,^28^ Clearly, more research is needed on this topic.

Africans and African-Americans are collectivistic population groups, who view the experience of social support from their extended families as important, and social support possibly has a protective effect on their cardiovascular status.^29^ Social support in the PC men though, contributed significantly to augmented CIMT (*r* = 0.53). PC has been implicated in depression. Therefore, it could be that these PC men had already reached avoidance, withdrawal and depression, to the point that social support became a stress factor, rather than a way of coping.^12^

Both hypotheses were accepted as, firstly, utilising an AC style was associated with increases in MS indicator values in urban African men. Secondly, a synergistic effect was illustrated in urban AC African men by positive associations, which existed between MS indicators and target end-organ damage depicted by carotid intima–media thickness and microalbuminuria. The SABPA study therefore indeed indicates that an AC style is associated with risk of pathology rather than being a health promoter.^3^

The SABPA study was limited with respect to the number of male participants. Enlarging the sample of participants would enhance identification of the influence of the specific coping styles on increased MS indicators and future development of the MS. The second recommendation is that seeking social support as a coping strategy should be further investigated, as well as its different effects in AC and PC men. Furthermore, indication of biological markers for smoking (Cotinin) and alcohol consumption (gamma glutamyl transferase) is essential. One last recommendation is to do a comparative study of Caucasian and African men utilising different coping styles, so as to show differences in population groups in accordance with development of the metabolic syndrome.

## Conclusion

Only BP and social support were associated with target endorgan damage in PC men, while in urban AC men, most MS indicators were positively associated with target end-organ damage, depicted by sub-clinical atherosclerosis and renal impairment.
